# The Functional Capacity of the Upper Airway in Older Adults with Chronic Stroke

**DOI:** 10.3390/geriatrics9060140

**Published:** 2024-10-31

**Authors:** Esther Prados-Román, Mónica Zapata-Soria, Irene Cabrera-Martos, Geraldine Valenza-Peña, Andrés Calvache-Mateo, Javier Martín-Núñez, Marie Carmen Valenza

**Affiliations:** Department of Physical Therapy, Faculty of Health Sciences, University of Granada, 18016 Granada, Spain; espra66@hotmail.com (E.P.-R.); monicazapatas@gmail.com (M.Z.-S.); geraldinevalenza@ugr.es (G.V.-P.); andrescalvache@ugr.es (A.C.-M.); javimn@ugr.es (J.M.-N.); cvalenza@ugr.es (M.C.V.)

**Keywords:** upper airway, stroke, respiration

## Abstract

(1) Background: Older adults with chronic stroke may experience compromised upper airway functions due to stroke-related changes and aging. This study aimed to evaluate the functional capacity of the upper airway in older adults with chronic stroke. (2) Methods: A total of 44 patients (22 in each group) were included in the study. The respiratory assessment involved measuring forced vital capacity, forced expiratory volume in one second, maximum voluntary ventilation, and peak cough flow. The voice assessment recorded intensity, frequency, shimmer, and the harmonics-to-noise ratio during a monologue task. Additionally, the maximum phonation time of /a/ and /s/ was recorded. The swallowing assessment included the Eating Assessment Tool—10 and the Swallowing Quality of Life questionnaire. (3) Results: Significant differences were found in the experimental group compared to the control group in maximum voluntary ventilation (44.59 ± 15.61 vs. 58.50 ± 28.08, *p* = 0.049) and peak cough flow (173.64 ± 101.09 vs. 291.59 ± 176.58, *p* = 0.009). Additionally, the experimental group showed poorer results than the control group in monologue intensity (66.60 ± 3.72 vs. 114.72 ± 63.09, *p* = 0.001), the harmonics-to-noise ratio (9.08 ± 2.06 vs. 10.26 ± 1.59, *p* = 0.042), and the maximum phonation time of /s/ (4.36 ± 1.67 vs. 8.09 ± 4.07, *p* < 0.001). Patients with stroke also had significantly lower values for swallowing efficiency and safety compared to the control group (7.05 ± 8.44 vs. 2.23 ± 4.14, *p* = 0.021) and reported poorer quality of life related to swallowing difficulties (185.50 ± 23.66 vs. 200.32 ± 19.60, *p* = 0.029). (4) Conclusions: Older adults with chronic stroke exhibited significantly reduced cough strength, voice intensity, maximum phonation time, and swallowing function compared to controls.

## 1. Introduction

Aging is a process of human life that involves changes in biological, psychological, and social areas [1]. The prevalence of diseases among older adults has generally increased over time [2]. Because of this, paying attention to the needs of individuals and early diagnosis of diseases is critical in this period of life. One of the most frequent and debilitating diseases in the global elderly population is stroke [3]. Although its consequences depend on the severity and site of the neurological injury, stroke survivors frequently experience medical complications, such as motor or cognitive impairment, and report worse functional outcomes [4,5]. Disturbances in the upper airway are common after stroke and include alterations in breathing control, respiratory mechanics, and breathing patterns, as well as difficulties related to speech and swallowing [6,7,8].

The upper airway is defined as the part of the respiratory system located between the nostrils or lips and the trachea. It plays a key role in respiration because it warms, humidifies, and filters the air that enters the lungs. Other relevant functions include speech and coordination of ventilation with swallowing as well as protection from aspiration of food through the cough reflex [9,10].

Aging has been associated with changes in the upper airway that include increased nasal resistance, parapharyngeal fat pad deposition, changes in sensation, and reduced reflex sensitivity [11]. In addition, age-related changes in upper airway muscles lead to an increase in the number of fast-fatigable fibers and reduced endurance [12]. Structural changes also include chest wall and thoracic spine deformities that affect chest wall compliance, leading to increased work of breathing. Additionally, respiratory muscle strength decreases with age and can impair effective cough [13]. Furthermore, increasing age is usually associated with ossification of the laryngeal skeleton, which can result in incomplete glottal closure with a negative impact on the protective functions of the larynx [14]. In older adults, the functional capacity of the upper airway can be affected by age-related dysfunction, but a stroke may lead to further compromise [6,7,8,11,12,13,14].

Upper airway dysfunction is a common but often under-recognized complication in older adults who have experienced a stroke. A stroke can disrupt the neural pathways that control respiratory, swallowing, and speech functions, exacerbating pre-existing age-related changes in the upper airway [6,7,8]. Older adults are already prone to decreased muscle tone and airway protection as well as impaired coordination between breathing and swallowing, making them especially vulnerable to further dysfunction after a stroke. Previous studies have shown that stroke patients over 50 years old exhibit reduced pulmonary function and respiratory pressures compared to the general older adult population [15,16]. They frequently report difficulty coughing effectively because of weakened respiratory muscles, which results from both neurological damage and the aging process. Consequently, this population is at increased risk for aspiration and respiratory infections such as pneumonia [15]. Additionally, older adults often develop dysphagia secondary to neurological and neurodegenerative disorders. Estupiñán Artiles et al. emphasized that early screening, identification, and management of dysphagia are important for preventing complications such as malnutrition, dehydration, and aspiration pneumonia in this population [17]. Although evidence is limited, previous studies have shown that stroke can also affect the voice, increasing the need for speech therapy in these patients [18,19]. 

Early identification and management of upper airway dysfunction in post-stroke patients are key to prevent complications and improve functionality and quality of life. Although previous studies have focused on changes related to aging or stroke, no studies have evaluated the functional aspects of the upper airway in older adults with stroke. Considering this, the purpose of this study was to evaluate the functional capacity of the upper airway through a respiratory, speech, and swallowing assessment in older adults with chronic stroke.

## 2. Materials and Methods

### 2.1. Study Design

A descriptive study was conducted. This study received ethical approval from the Andalusian Biomedical Research Ethics Committee, project ICTUSRESPIRA. Before being included in the study, patients were informed about the purpose and procedure of the study and gave written informed consent to participate (Supplementary File S1). The protocol complied with the standards for human experiments set by the Declaration of Helsinki.

### 2.2. Study Participants

Participants were recruited from two associations: Asociación Granadina de familias por la Rehabilitación del Daño Cerebral adquirido (AGREDACE) and the Asociación de familiares y enfermos de ictus de Granada (Neuro-Afeic) (Granada, Spain). These non-profit organizations aim to provide information and support to individuals affected by acquired brain injuries and their families. The activities they engage in include raising public awareness about the disease, coordinating healthcare services, securing resources, and providing specialized care such as physical rehabilitation and psychological support to both patients and their families. In addition, a control group of healthy controls matched by age and gender with the same exclusion criteria was included. Controls were recruited from the community.

The inclusion criteria were as follows: patients aged 65 and over with a clinical diagnosis of stroke and ≥6 months’ duration since stroke diagnosis.

The exclusion criteria were persons with a central nervous system disease other than stroke or multiple strokes, cardiopulmonary disease associated with decreased functional capacity, co-occurring aphasia, severe cognitive impairment (Montreal Cognitive Assessment [MoCA] < 26 points) [20], tracheostomy, prolonged orotracheal intubation (>48 h), presence of cancer, and absence of neuromotor competence to carry out the respiratory function tests. Patients with recent otorhinolaryngological, abdominal, or thoracic surgery were also excluded.

### 2.3. Procedure

The assessment was conducted in a laboratory by a blinded assessor. Descriptive measures included age, gender, body mass index, disease duration, type of stroke, and disability and health-related quality of life after stroke, evaluated using the Stroke Impact Scale [21]. The main outcome measures included a respiratory, voice, and swallowing assessment.

#### 2.3.1. Respiratory Assessment

The respiratory assessment included a spirometry test and a cough assessment. The parameters evaluated through the spirometry test were forced vital capacity (FVC), forced expiratory volume in one second (FEV1), and maximum voluntary ventilation (MVV). Cough effectiveness was assessed by measuring peak cough flow (PCF).

The spirometry test was performed with a Spirobank-II handheld spirometer (Medical International Research USA, Inc.; New Berlin, WI, USA) and WinspiroPRO^®^ software 7.9.0. Participants were sitting in a chair with armrests and had their feet flat on the floor and legs uncrossed during the assessment. They were asked to take a maximal inspiration and then to exhale as forcefully, quickly, and fully as they could into a spirometer, which records the volume of air over time. FVC refers to the maximal volume of air exhaled with maximally forced expiratory effort and FEV1 is the maximal volume of air exhaled in the first second of forced expiration. MVV is the maximum volume of air a subject can breathe as rapidly and deeply as possible over a specified time. It is influenced by airway resistance, respiratory muscle function, ventilation control mechanisms, and the compliance of both the lungs and chest wall. Participants were instructed to breathe as forcefully, quickly, and deeply as possible for 12–15 s into a spirometer. Normal findings for these variables are those above 80% of the predicted values [22]. Patients were asked to perform three trials, using the best value of each outcome for further analysis. The lung function values were expressed as a percentage of the predicted values according to the age, gender, height, and weight of the participant.

PCF (L/min) shows the effectiveness of secretion clearance and was assessed with a peak flow meter (Micro Medical, Cambridge, UK). The patient was asked to inspire fully and then cough forcibly through the mouthpiece. Three trials were performed, and the average was considered for the analysis. Effective peak cough flow in healthy subjects exceeds values of 360 to 400 L/min [23]. Effective coughing is affected if the peak cough flow value is less than 170 L/min. Values lower than 270 L/min are associated with increased secretion retention and risk of infection [24].

#### 2.3.2. Voice Assessment

The voice assessment included a monologue task analysis through voice recordings and the maximum phonation time of vowel /a/ and consonant /s/.

For the monologue task, participants were asked to talk about a topic of their choice for at least 60 s without long pauses or examiner questions. Speech samples were recorded. Before the monologue, the subjects were given topic suggestions such as family, school, sports, friends, hobbies, or films. The analysis was conducted using PRAAT software (version 5.1.05) [25]. The outcomes obtained in the analysis included mean intensity (decibels), mean frequency (hertz), shimmer (i.e.; the variability of the maximum extent of the amplitude of each vocal vibration from one cycle to the next), and the harmonics-to-noise ratio (i.e.; the amount of noise in voice signals, in decibels) [26].

For the maximum phonation time (MPT) assessment, participants were instructed to take a deep inhalation and perform a sustained phonation of vowel /a/ and consonant /s/ in the most comfortable pitch and loudness as long as possible. MPT for each participant was measured 3 times with a stopwatch, and the highest value obtained was considered for the analysis [27].

#### 2.3.3. Swallowing Assessment

For the swallowing assessment, the patients were asked to complete the 10-Item Eating Assessment Tool (EAT-10) and the Swallowing Quality of Life (SWAL-QOL) questionnaire. 

The EAT-10 is a self-administered questionnaire for dysphagia screening, with 10 items ranging from 0 (lack of symptoms) to 4 (severe problem). An EAT-10 score ≥ 3 is abnormal and indicates the presence of swallowing difficulties. The EAT-10 has been confirmed to have excellent internal consistency, test–retest reproducibility, and criterion-based validity [28].

The SWAL-QOL is a self-administered questionnaire used to evaluate the impact of dysphagia symptoms on daily living. The questions are designed to capture the individual’s experience with swallowing difficulties over the past month [29]. This questionnaire contains 44 items grouped into 11 subscales including burden, eating duration, eating desire, frequency of symptoms, food selection, fear of intake, mental health, social functioning, communication, sleep, and fatigue. Participants are asked to report the frequency of each statement within each domain. In the burden subscale, they are asked to rate how difficult it is for them to cope with their swallowing problem and whether it is a significant concern in their life. The eating duration subscale assesses whether they take longer to eat compared to others. The appetite subscale gathers information on whether participants care about eating, if they enjoy eating, or if they are rarely hungry. The frequency of symptoms includes issues such as coughing, choking, drooling, and food residue. The food selection subscale explores difficulties in finding foods that they both like and can eat. Communication addresses problems with being understood while speaking and difficulties in speaking clearly. The fear of intake subscale includes concerns about getting pneumonia or choking while eating or drinking, and the mental health subscale evaluates the presence of depression, irritation, annoyance, frustration, or sadness related to swallowing issues. This questionnaire also covers social aspects, including limitations going out, participating in social activities, or engaging in usual activities due to swallowing problems. Finally, the fatigue and sleep subscales inquire about feelings of tiredness and difficulties falling and staying asleep. Each item is scored on a Likert scale ranging from 1 (worse condition) to 5 (better condition), and the overall score ranges from 0 to 100. Lower scores are related to a poor quality of life. This questionnaire is valid and reliable [29].

Further information concerning self-administered questionnaires can be found in Supplemental File S2.

### 2.4. Statistical Analysis

Descriptive statistics were expressed as the mean (standard deviation) in non-categorical variables and the number of cases in categorical variables. Descriptive data and clinical assessment results were compared by means of *t*-tests using the Statistical Package for the Social Sciences (IBM Corp. Released 2011. IBM SPSS Statistics for Windows, version 20.0. Armonk, NY, USA: IBM Corp). Before the statistical analysis, the Kolmogorov–Smirnov test was performed to assess the normality of continuous data. The level of significance was set at *p* < 0.05.

The sample size was calculated using GPower^®^ software, version 3.1.9.2 (Franz Faul, Universitat Kiel, Kiel, Germany).. The effect size was 0.89 for the PCF measure. The sample size calculation required 21 patients in each group with a 0.05 alpha error and a study power of 80%. Anticipating a dropout of 10%, a sample size of 48 participants was needed.

## 3. Results

### 3.1. Descriptive Characteristics of the Participants

A total of 44 patients were finally included in the study. Table 1 shows the characteristics of the population studied. There were no significant differences in gender, age, or body mass index between groups. The mean duration of the stroke was 40.27 ± 28.50 months.

### 3.2. Respiratory Assessment

The respiratory assessment is included in Table 2. Significant between-group differences were found in MVV and peak cough flow, with better results in the control group. No significant between-group differences were found in the FVC and FEV_1_ values, although in FEV_1_, the *p*-value was 0.05.

### 3.3. Voice Assessment

The voice assessment is included in Table 3. In the monologue task, the control group showed similar values in the frequency and shimmer parameters, showing no significant differences in the amplitude of the sound wave or the intensity of the vocal output. Moreover, the maximum phonation time of vowel /a/ was similar in both groups. The between-group analysis revealed significant differences (*p* < 0.05) in the intensity and mean harmonics-to-noise ratio during the monologue task, showing better signal quality in the control group, and in the maximum phonation time of consonant /s/.

### 3.4. Swallowing Assessment

The swallowing assessment values are presented in Table 4. Significant between-group differences were found in the EAT-10 score, with the experimental group showing more swallowing difficulties. In addition, participants included in the experimental group showed worse values in the SWAL-QOL total score, specifically in the burden and symptom frequency subscales. The remaining subscales showed no significant differences between the groups, including selecting food they like and can eat, the presence of symptoms that affect mental health, and perceived fatigue.

## 4. Discussion

The purpose of this study was to evaluate the functional capacity of the upper airway through a respiratory, speech, and swallowing assessment in older adults with chronic stroke. Results showed that some specific aspects related to the functional capacity of the upper airway were impaired in older adults with chronic stroke in comparison to healthy controls. Specifically, MVV and PCF were significantly worse in the respiratory assessment. In addition, the intensity and mean harmonics-to-noise ratio during the monologue task and the maximum phonation time of consonant /s/ were reduced in the voice assessment. Finally, swallowing difficulties and reduced quality of life related to these difficulties were also found in the swallowing assessment.

The upper airway involves a sophisticated and complex control system requiring neuromuscular coordination to subserve the functions of respiration, speech, and swallowing [10]. Although no significant changes were found in the FVC and FEV_1_ values, the results of the assessment were lower in the group of older adults with stroke. The FVC decrease in older adults is related to an increased residual volume and decreased diffusion capacity [30]. Our results showed that PCF was lower in older adults with stroke. An effective cough is needed to avoid respiratory tract infections, which are a frequent cause of hospital admission in the elderly population [31]. In this regard, an early assessment and a therapeutic approach are key to preventing respiratory complications in older adults with stroke. MVV was also lower in the experimental group. Stroke is related to a decrease in diaphragmatic strength that causes muscle atrophy and a decrease in fast-twitch fibers. Aging is also related to a decline in diaphragmatic strength that may predispose elders to diaphragmatic fatigue and ventilatory failure during increased ventilatory load [32]. In fact, older adults with stroke may present worse values in this variable. 

In our study, the voice assessment showed that intensity, the mean harmonics-to-noise ratio during a monologue task, and the maximum phonation time of consonant /s/ were significantly worse in the older adults with stroke evaluated. Communication is frequently impaired in patients with stroke, including aphasia and speech difficulties such as disruptions in speech motor planning and execution processes. The study by De Cock et al. showed that the most frequent disorders in speech were the imprecise articulation of consonants, harsh voice quality, and audible inspiration [33]. In our results, the values for the maximum phonation time of consonant /s/ were worse than those for vowel /a/. Compared to the maximum phonation time values obtained by Rees et al.; our results were shorter in both groups [34]. Different changes related to aging impact the voice, including thinner elastin fibers of lamina propria and a reduction in mucous production. Functional changes include poor voice quality, vocal tension, tremor, and altered frequency [27]. Different factors have been associated with voice disorders among older adults, including physical and psychosocial aspects such as colds, bronchitis, asthma, anxiety, and depression [35]. Some areas such as voice-related effort and discomfort negatively impact the quality of life of patients [36].

Both speech and swallowing involve oral, lingual, laryngeal, and respiratory control. This control allows vibration of the vocal folds for voice production and contraction of the aryepiglottic folds to avoid the aspiration of substances into the trachea [37]. Our results showed that swallowing was impaired significantly in older adults with stroke compared to healthy controls. The prevalence of swallowing disorders in older adults ranges from 10 to 60% and is often underestimated [38]. Aging involves a reduction in the elasticity of the oral mucosa, less production of saliva, decreased muscle function, and reduced mobility of orofacial structures. Older adults with stroke frequently develop oropharyngeal dysphagia, with changes in the swallowing procedure such as difficulty in chewing, increased oral preparation time, and inability to push the bolus into the pharynx [39]. They also frequently experience episodes of laryngeal penetration or bronchial aspiration [40]. Similarly to our study, Pontes et al. [41] evaluated quality of life related to dysphagia in older adults with stroke. They found a negative impact of swallowing disturbances on quality of life. Specifically, they found that an increase in feeding time and selection of food were the subscales most related to poor quality of life [41]. In our results, burden and symptom frequency were the subscales with significantly worse results compared to controls.

Some limitations of our study must be acknowledged. Participants were recruited from the same two associations, which could result in potential selection bias. In addition, the sample was small and heterogeneous, given that the clinical profile of patients with stroke depends on the severity and site of the neurological injury. Another limitation is that only some tools were selected for functional capacity assessment. Other respiratory evaluations could also be of interest, such as polysomnography, considering that a decrease in upper airway lumen or in genioglossus muscle strength, the descent of the hyoid bone, and the lengthening of the pharyngeal airway may alter airway stability, increase airway resistance, and predispose individuals to airway collapse with aging [42]. Considering this, future studies should include a comprehensive evaluation of sleep patterns and quality. Additionally, swallowing function was not assessed with an objective measure such as a videofluoroscopy. Furthermore, changes in the upper airway may affect exercise capacity. Hence, it would be interesting to assess patients’ ability to perform dynamic exercise, which should be reduced in older adults and patients with stroke.

The findings of this study have significant clinical implications. The prevalence of comorbidities increases with age, and stroke may exacerbate the development of upper airway disturbances. Evaluating and understanding upper airway function can substantially improve patient care. A routine assessment of the functional capacity of the upper airway could be relevant among older adults with stroke to provide an early diagnosis, adequate treatment, and improvement in quality of life. In addition, it would enable the identification of patients at risk for complications such as aspiration, airway obstruction, and respiratory infections, which often arise from muscle weakness and coordination issues following a stroke.

Early identification of these risks would allow for prompt and targeted interventions. In fact, a comprehensive evaluation of upper airway function supports the development of personalized treatment plans that address respiratory, communicative, and swallowing functions. The follow-up of patients with chronic stroke provides critical insights into patient progress and the effectiveness of rehabilitation efforts, which helps prevent long-term complications. To achieve optimal patient management, a multimodal approach is essential and further research is needed to define these strategies. Incorporating early assessment of upper airway function into routine clinical practice would facilitate accurate risk assessment, tailored treatment planning, and effective monitoring of older adults with chronic stroke.

## 5. Conclusions

Our results show that some aspects related to the functional capacity of the upper airway are impaired in older adults with stroke. Specifically, such patients presented worse values in PCF, MVV, voice intensity, maximum phonation time of a consonant, and swallowing parameters compared to controls. However, a larger study is required to validate the results of this study. A routine assessment of the functional capacity of the upper airway could be relevant among older adults with stroke to provide an early therapeutic approach.

## Figures and Tables

**Table 1:** Descriptive characteristics of the sample.

Variables	Experimental Group (*n* = 22)	Control Group (*n* = 22)	*p*-Value
Gender (n females)	9	9	-
Age (years) (mean ± SD)	72.18 ± 7.33	68.77 ± 3.46	0.055
BMI (kg/m^2^) (mean ± SD)	27.19 ± 3.66	28.78 ± 4.84	0.390
Illness duration (months) (mean ± SD)	40.27 ± 28.50	-	-
Type of stroke, n (% ischemic stroke)	17 (77.3)		
Stroke Impact Scale score (mean ± SD)	51.92 ± 16.21		

BMI: body mass index; SD: standard deviation.

**Table 2:** Results of the respiratory assessment.

Variables	Experimental Group (*n* = 22)	Control Group (*n* = 22)	*p*-Value
Spirometric parameters (mean ± SD)			
FVC (%)	67.77 ± 23.94	74.00 ± 26.83	0.421
FEV_1_ (%)	70.09 ± 25.00	84.05 ± 20.80	0.050
MVV (%)	44.59 ± 15.61	58.50 ± 28.08	0.049
Cough (mean ± SD)			
PCF (L/min)	173.64 ± 101.09	291.59 ± 176.58	0.009

FEV_1_: forced expiratory volume in one second; FVC: forced vital capacity; MVV: maximum voluntary ventilation; PCF: peak cough flow; SD: standard deviation.

**Table 3:** Results of the voice assessment.

Variables	Experimental Group (*n* = 22)	Control Group (*n* = 22)	*p*-Value
Monologue task (mean ± SD)			
Intensity (dB)	66.60 ± 3.72	114.72 ± 63.09	0.001
Frequency (Hz)	182.07 ± 52.79	168.21 ± 42.42	0.347
Shimmer	1.26 ± 0.19	1.19 ± 0.15	0.169
Mean harmonics-to-noise ratio (dB)	9.08 ± 2.06	10.26 ± 1.59	0.042
Maximum phonation time (mean ± SD)			
Vowel /a/ (s)	10.79 ± 5.27	13.02 ± 6.42	0.221
Consonant /s/ (s)	4.36 ± 1.67	8.09 ± 4.07	*p* < 0.001

SD: standard deviation.

**Table 4:** Results of the swallowing assessment.

Variables	Experimental Group (*n* = 22)	Control Group (*n* = 22)	*p*-Value
EAT-10 (mean ± SD)	7.05 ± 8.44	2.23 ± 4.14	0.021
SWAL-QOL questionnaire (mean ± SD)			
Burden	8.18 ± 2.08	9.91 ± 0.43	*p* < 0.001
Eating duration	7.68 ± 3.34	9.05 ± 2.40	0.128
Eating desire	11.23 ± 3.65	12.68 ± 2.97	0.154
Symptom frequency	59.09 ± 10.49	67.86 ± 4.68	0.001
Food selection	8.86 ± 1.49	9.14 ± 1.58	0.559
Communication	8.68 ± 1.91	9.41 ± 1.53	0.171
Fear of intake	14.64 ± 5.38	15.86 ± 6.99	0.517
Mental health	22.18 ± 2.61	22.96 ± 3.47	0.409
Social functioning	24.41 ± 1.14	22.91 ± 4.02	0.100
Fatigue	12.91 ± 2.62	12.36 ± 4.73	0.638
Sleep	7.64 ± 2.70	8.18 ± 2.59	0.498
Total score	185.50 ± 23.66	200.32 ± 19.60	0.029

EAT-10: 10-Item Eating Assessment Tool; SWAL-QOL: Swallowing Quality of Life questionnaire; SD: standard deviation.

## Data Availability

Data are available on request due to restrictions.

## Supplementary Materials

The following supporting information can be downloaded at: https://www.mdpi.com/article/10.3390/geriatrics9060140/s1. File S1: Written informed consent model; File S2: Self-administered questionnaires information. References [43,44] are citied in the Supplementat Materials.
