# Supplementary material for: The Functional Capacity of the Upper Airway in Older Adults with Chronic Stroke
Source: Geriatrics (Basel). 2024 Oct 31;9(6):140. doi: 10.3390/geriatrics9060140 (PMC11587141; doi:10.3390/geriatrics9060140)
Supplement: Supplementary file 1 [file geriatrics-09-00140-s001.zip › geriatrics-3051863-supplementary.pdf]

Supplemental file S1_ CONSENTIMIENTO INFORMADO

Título del Proyecto: Evaluación funcional de vía aérea superior en adultos mayores con ictus crónico

Apellidos y nombre del/de la participante en el estudio:

DNI / NIE:

Apellidos y nombre del/de la representante legal:

DNI / NIE:

CONSENTIMIENTO:

Yo, D./Dña. declaro bajo mi responsabilidad que **he leído y comprendido la hoja de Información**, de la que se me ha entregado un ejemplar.

He **recibido suficiente información** sobre mi participación en el proyecto, sobre la utilización de mis datos personales e información asociada. He podido hacer preguntas sobre la información recibida y hablar con el profesional indicado, quien me ha resuelto todas las dudas que le he planteado.

- Comprendo que mi participación es voluntaria.
- Comprendo que puedo retirarme del estudio cuando quiera sin dar explicaciones.

Doy mi consentimiento para que los datos clínicos sean tratados por el investigador principal y el resto de colaboradores.

Doy mi consentimiento para que los investigadores puedan ponerse en contacto conmigo en caso necesario.

Indique el medio de hacerlo:
☐ Teléfono:
☐ Correo electrónico:
☐ Otros: (identificar)

Sé que puedo **revocar**, en cualquier momento, el consentimiento otorgado en este documento.
En , a de de

PARTICIPANTE REPRESENTANTE LEGAL
(sólo en caso de incapacidad del participante)
Fdo.: Fdo.:

PROFESIONALES QUE INTERVIENEN EN EL PROCESO DE INFORMACIÓN Y/O CONSENTIMIENTO

Los siguientes profesionales declaran que se ha explicado la información relativa a la participación en el proyecto

Fecha:
Investigador:

NORMATIVA APLICABLE
- Ley 14/2007, de 3 de julio, de Investigación biomédica.
- Ley 41/2002, de 14 de noviembre, básica reguladora de la autonomía del paciente y de derechos y obligaciones en materia de información y documentación clínica.
- Real Decreto 1716/2011, de 18 de noviembre, por el que se establecen los requisitos básicos de autorización y funcionamiento de los biobancos con fines de investigación biomédica y del tratamiento de las muestras biológicas de origen humano, y se regula el funcionamiento y organización del Registro Nacional de Biobancos para investigación biomédica.
- REGLAMENTO (UE) 2016/679 DEL PARLAMENTO EUROPEO Y DEL CONSEJO de 27 de abril de 2016 relativo a la protección de las personas físicas en lo que respecta al tratamiento de datos personales y a la libre circulación de estos datos y por el que se deroga la Directiva 95/46/CE (Reglamento general de protección de datos)
- Ley Orgánica 3/2018, de 5 de diciembre, de Protección de Datos Personales y garantía de los derechos digitales del participante

Supplemental file S2

Self-administered questionnaires:

- 10-Item Eating Assessment Tool (EAT-10):

The EAT-10 is a self-administered questionnaire for dysphagia screening, with 10 items ranging from 0 (lack of symptoms) to 4 (severe problem). An EAT-10 score ≥ 3 is abnormal and indicates the presence of swallowing difficulties.

The version used in this study can be accessed at:

- Burgos R, Sarto B , Segurola H , Romagosa A , Puiggrós C , Vázquez C , Cárdenas G , Barcons N , Araujo K , Pérez-Portabella C. Translation and validation of the Spanish version of the EAT-10 (Eating Assessment Tool-10) for the screening of dysphagia. Nutricion Hospitalaria 2012; 27(6):2048-2054. doi: 10.3305/nh.2012.27.6.6100

- Swallowing quality of life (SWAL-QOL) questionnaire:

The SWAL-QOL is a self-administer questionnaire used to evaluate the impact of dysphagia symptoms on daily living. It contains 44 items grouped in 11 subscales including burden, eating duration, eating desire, frequency of symptoms, food selection, fear of intake, mental health, social functioning, communication, sleep, and fatigue.

The Spanish version used in this study can be accessed at:

- Zaldibar-Barinaga MB, Miranda-Artieda M, Zaldibar-Barinaga A, Pinedo-Otaola S, Erazo-Presser P, Tejada-Ezquerro P. Spanish version of the Swallowing Quality of Life Questionnaire (SWAL-QoL): Initial phase of cross-cultural adaptation. Rehabilitación 2013; 47 (3):136-140. doi: 10.1016/j.rh.2013.03.002.
